# Competition for engineering tenure-track faculty positions in the United States

**DOI:** 10.1093/pnasnexus/pgae169

**Published:** 2024-05-07

**Authors:** Siddhartha Roy, Brenda Velasco, Marc A Edwards

**Affiliations:** Department of Environmental Sciences, Rutgers University, New Brunswick, NJ 08901, USA; Civil and Environmental Engineering, Virginia Tech, Blacksburg, VA 24061, USA; Civil and Environmental Engineering, Virginia Tech, Blacksburg, VA 24061, USA; Civil and Environmental Engineering, Virginia Tech, Blacksburg, VA 24061, USA

**Keywords:** academic jobs, engineering education, environmental engineering, graduate education, science and technology workforce

## Abstract

How likely are engineering PhD graduates to get a tenure-track faculty position in the United States? To answer this question, we analyzed aggregated yearly data on PhD graduates and tenure-track/tenured faculty members across all engineering disciplines from 2006 to 2021, obtained from the American Society of Engineering Education. The average likelihood for securing a tenure-track faculty position for engineering overall during this 16-year period was 12.4% (range = 10.9–18.5%), implying that roughly 1 in 8 PhD graduates attain such positions. After a significant decline from 18.5 to 10.9% between 2006 and 2014 (*R*^2^ = 0.62; *P* < 0.05), a trend consistent with a period of rising competition, the outlook has since stabilized between 11.3 and 12% (*R*^2^ = 0.04; *P* > 0.05). Given that most engineering PhD graduates will never secure a tenure-track faculty position, emphasizing alternative career tracks during doctoral training could align expectations better with reality.

A Doctor of Philosophy (PhD) degree is a prerequisite for a tenure-track (TT) faculty position. Over the past five decades, the percentage of full-time faculty positions in US universities has steadily declined (1), while the production of science and engineering PhD graduates has nearly doubled (2). We assume that this asymmetry creates increased competition for PhD graduates interested in getting TT positions (3, 4).

To quantify this effect, Larson et al. (3) applied the basic reproductive number (*R*_0_) concept from epidemiology to estimate the average number of PhD graduates each faculty member “births” over their academic career. An exemplary *R*_0_ of 1 indicates that each professor replaces themselves by graduating just 1 PhD student in a career, which implies that the likelihood of a PhD graduate getting a TT position is 1 in 1 or 100% assuming a steady number of faculty positions. Using this approach, the academic *R*_0_ for the entire field of engineering was calculated to be 7.8 (range = 1.0–19.0) in 2011 and the corresponding likelihood of securing a faculty position was 1 in 7.8 or 12.9% (range = 5.7–100%). Here, we update Larson et al.’s analysis to the 16-year period of 2006–2021 to examine trends in the crude academic *R*_0_ and the associated likelihood of engineering PhD graduates getting TT positions.

We sourced aggregated yearly data on PhD graduates and TT/tenured faculty members for all engineering departments in the United States (Table 1) from 2006 to 2021 from annual reports of the American Society of Engineering Education (ASEE) (5). We removed 2019 data from our analysis because of serious data anomalies (Text S1); the total TT/tenured faculty counts for engineering overall and certain disciplines were much higher (and sometimes more than twice as much) compared with both prior and later years, which is impossible. We estimated the crude academic *R*_0_, i.e. the number of PhD graduates per faculty member over an average career duration of 20 years (6) using Eq. 1. Assuming a steady state in the number of TT/tenured faculty positions available (3), the inverse of the academic *R*_0_ approximates the likelihood of engineering PhD graduates themselves getting a TT position (hereafter referred to as “likelihood”; Eq. 2).


(1) $$\mathrm{Crude}\;\mathrm{academic}\;\mathrm{reproductive}\;\mathrm{number}\;(R_{0})=\;\frac{\mathrm{PhD}\;\mathrm{graduates}}{\mathrm{Tenured}\;\mathrm{or}\;\mathrm{tenure}\;\mathrm{track}\;\mathrm{faculty}}\times 20$$

(2) $$\mathrm{Likelihood}\;\mathrm{of}\;\mathrm{PhD}\;\mathrm{graduates}\;\mathrm{getting}\;a\;\mathrm{TT}\;\mathrm{position}=\;\frac{1}{R_{0}}$$

(3) $$\mathrm{Likelihood}\;\mathrm{of}\;\mathrm{PhD}\;\mathrm{graduates}\;\mathrm{not}\;\mathrm{getting}\;a\;\mathrm{TT}\;\mathrm{position}=1-\;\frac{1}{R_{0}}$$

**Table 1.** Rough proportion of engineering PhD graduates (averaged every 4 years) who would not secure a TT faculty position in their own field in the United States, 2006–2021.

No.	Discipline	2006–2009 (%)	2010–2013 (%)	2014–2017 (%)	2018–2021^a^ (%)
a	All	85.7	87.3	88.5	88.5
b	Aerospace	86.9	89.2	90.1	91.4
c	Architectural	23.2	19.0	78.2	65.3
d	Biological and Agricultural	71.6	82.4	83.4	80.8
e	Biomedical	90.9	92.4	92.0	91.6
f	Chemical	89.3	89.0	90.3	90.2
g	Civil	80.5	82.6	85.7	88.3
h	Civil/Environmental	67.3	67.9	78.1	75.4
i	Computer Science (inside engineering)	85.8	86.8	87.2	87.4
j	Computer Science (outside engineering)	85.5	87.0	88.4	87.9
k	Electrical/Computer	75.7	75.2	81.8	84.1
l	Engineering (general)	74.3	73.7	74.5	75.5
m	Engineering Management	82.5	90.4	91.1	93.7
n	Engineering Science and Engineering Physics	88.6	90.8	89.2	89.1
o	Environmental	92.6	94.3	95.8	93.0
p	Industrial/Manufacturing/Systems	84.6	85.9	87.2	89.0
q	Mechanical	80.8	82.5	84.7	85.5
r	Metallurgical and Materials	93.0	93.8	94.1	93.1
s	Mining	67.5	61.1	82.5	84.6
t	Nuclear	91.1	92.9	94.5	93.6
u	Other	83.4	85.8	89.2	89.3
v	Petroleum	88.2	90.4	91.2	93.2
w	Civil/Environmental composite (g + h + o)	81.4	82.8	85.9	85.6

^a^Excluding 2019 data because of data discrepancies (Text S1).

The average academic *R*_0_ for engineering overall was 8.1 (range = 5.4–9.1) during 2006–2021, which is slightly higher but relatively stable in recent years (Fig. 1). The academic *R*_0_ increased significantly at an average rate of 8.6% per year from 2006 (*R*^2^ = 0.72; *P* < 0.05). However, from 2015 to 2021, the change was insignificant at −0.3% (*R*^2^ = 0.05; *P* > 0.05). This translates to a 1 in 8.1 or 12.4% average likelihood for getting a TT position (range = 10.9–18.5%). After a significant decline from 18.5 to 10.9% between 2006 and 2014 (*R*^2^ = 0.62; *P* < 0.05), a trend consistent with a period of rising competition, the likelihood has since stabilized between 11.3 and 12% (*R*^2^ = 0.04; *P* > 0.05). The observed trend in the proportion of all engineering PhDs not getting faculty positions in their field in the United States (Eq. 3) also illustrates a slight increase followed by a plateau over the 16-year period (Table 1).

**Fig. 1.**
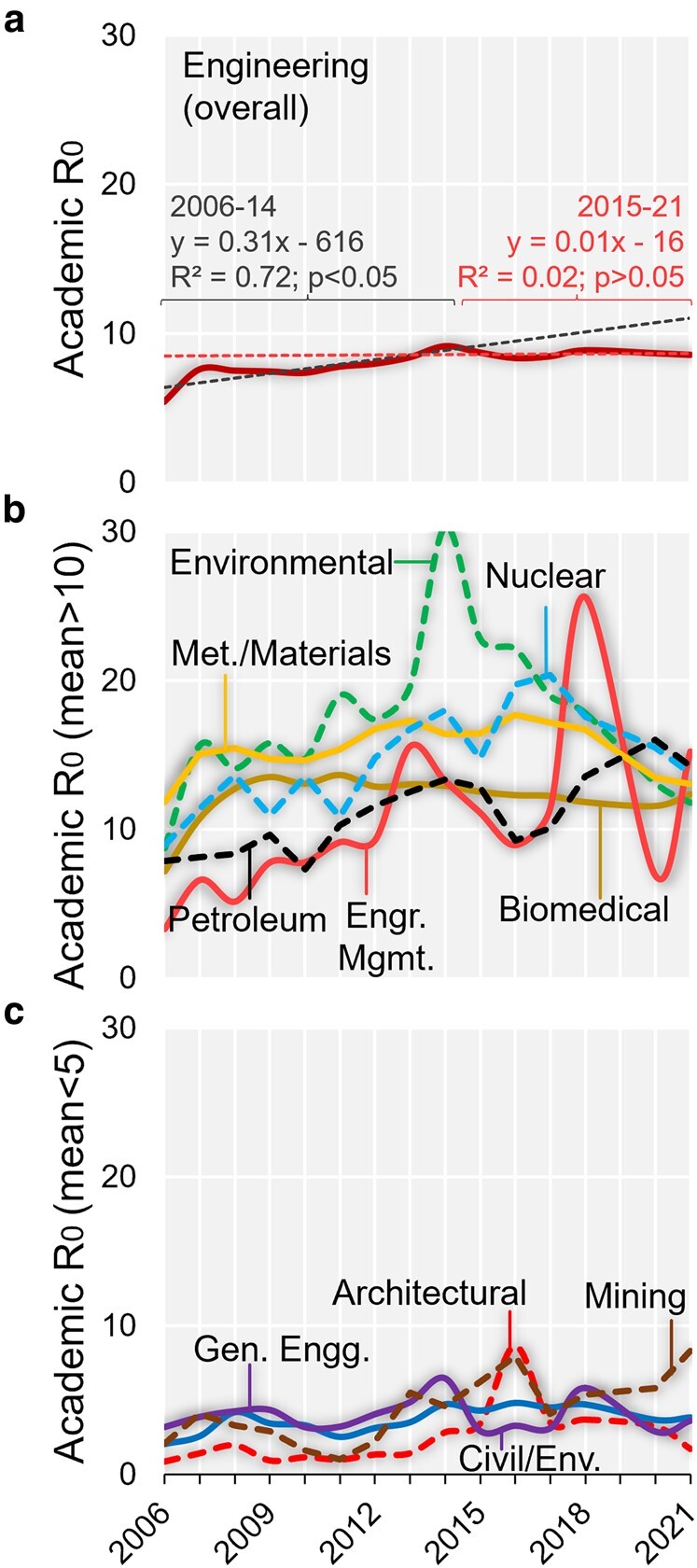
Trends in academic reproductive number (*R*_0_) for (a) engineering overall, (b) engineering disciplines with average *R*_0_ > 10, and (c) engineering disciplines with average *R*_0_ < 5, in the United States, 2006–2021. (Excluding 2019 data because of discrepancies; Text S1.) For overall engineering, the academic *R*_0_ from 2006 was increasing significantly at an average rate of 8.6% or 0.31 per year, but from 2015 to 2021, the change was insignificant (−0.3%).

There was substantial variability in the average academic *R*_0_ between engineering disciplines. The average academic *R*_0_ was 10 or higher (i.e. <10% likelihood) for Engineering Management (10.8), Petroleum (11.2), Biomedical (12.1), Nuclear Engineering (14.8), Metallurgical and Materials (15.4), and Environmental Engineering (17.3) during 2006–2021. Furthermore, the average academic *R*_0_ was below 5 (or >20% likelihood) for Architectural (2.5), Civil/Environmental (3.7), General Engineering (3.9), and Mining (4.5) over the same period. Finally, the average academic *R*_0_ for the remaining engineering disciplines (Table 1) was in the 5–10 range, which equates to a rough likelihood between 10 and 20%.

At first glance, Environmental Engineering appears to be the field with the toughest competition, with the highest average academic *R*_0_ across all disciplines. Environmental Engineering's academic *R*_0_ rose post-2010, peaking in 2014 before dropping to pre-2010 levels in 2020–2021 (Fig. 1), suggesting an increased availability and hiring of faculty positions and/or a lower count of PhD graduates per faculty in recent years. However, because some universities can report their Environmental Engineering data to the “Civil/Environmental Engineering” category, the true academic *R*_0_ could be lower than the current estimate. In fact, when we combined data for “Civil” (*R*_0_ = 6.6), “Civil/Environmental” (*R*_0_ = 3.7), and “Environmental” (*R*_0_ = 17.3), the average academic *R*_0_ was 6.3.

When comparing the COVID-19 pandemic period (2020–2021) with the prepandemic years (2015–2018), the academic *R*_0_ for engineering overall did not change (+0.2%). The average academic *R*_0_ rose (≥20%) for Civil, Industrial/Manufacturing/Systems, and Petroleum Engineering and dropped (≥20%) for Architectural, Engineering Management, Environmental, and Metallurgical/Materials Engineering. A visual examination (Fig. 1), however, revealed that the normal statistical variation in academic *R*_0_ was preserved from 2015 to 2018 into the COVID-19 era for Civil, Environmental, Industrial/Manufacturing/Systems, and Petroleum Engineering. Metallurgical and Materials Engineering alone witnessed an abrupt drop to pre-2010 levels. (The underlying data on total PhD students for Architectural Engineering and Engineering Management are low [sometimes <20 or <100], sometimes generating unreliable academic *R*_0_, making between-periods comparisons difficult.) Overall, it is too early to state whether the pandemic has had a definitive impact in competition for engineering faculty positions.

Nearly 80% of TT faculty members hired during 2011–2020 were trained at just 20% of US universities, demonstrating a “prestige hierarchy” with a lower likelihood of PhD graduates from the less prestigious universities getting permanent faculty positions (7). The ever-increasing competition may also help explain why National Science Foundation graduate research fellows in engineering (*n* = 244) and graduate students generally believe that pressures for funding, publications, and tenure are among the biggest drawbacks of academia (8, 9). An overemphasis on quantitative metrics can increase perverse incentives for academics and threaten scientific integrity (10).

Paradoxically, recent surveys have found that the research metrics of funding, publication count, and journal impact factors cannot fully explain why some applicants receive job offers, while others do not (11, 12). This might be attributed to increased emphasis on diversity, equity, and inclusion in US faculty hiring over the past 5–10 years (13). Overall, about 33% of Physical Sciences and Engineering postdoctoral researchers find a TT position within 5–6 years of graduating with a PhD (14). The hiring of new faculty members is also impacted by established professors retiring much later following the repeal of the mandatory retirement policy (at age 70) in 1994 (15).

These relatively steady results over the last 10 years or so suggest that competition for academic jobs may be stabilizing and reaching a “new normal.” Since the majority of PhD graduates will never secure a TT faculty position (see Table 1 in Ref. (14)), there has been a corresponding shift in career aspirations, especially following the COVID-19 pandemic, with less than half of graduate students reporting in a recent *Nature* survey (*n* = 3,253) that they want a long-term career in academia (8). A major barrier for STEM PhD students is a lack of knowledge about alternative academic careers and the training and networks needed to find them (16). The alternative academic career tracks (e.g. government and industry scientists (14, 17)) could be emphasized more during PhD training to ensure that expectations more aptly match the realities of TT searches.

Our study has limitations. Some universities might report their numbers from the same engineering discipline to different categories under ASEE guidelines, which can affect aggregate counts for PhD graduates and faculty for the engineering categories and our calculations. We discussed this issue explicitly for the Civil, Civil/Environmental, or Environmental categories, but it may also apply to Electrical and Computer Engineering and other groupings. The calculations for engineering overall (“All” in Table 1) do not include the nonengineering category of Computer Science (outside engineering) that the ASEE also compiles data for. Trends for the ASEE engineering categories of Electrical and Computer are not presented herein because the TT/tenured faculty counts were not reported for certain years in the source ASEE reports. Our findings may be less reliable for certain fields like Architectural Engineering, Engineering Management, and Mining Engineering because small changes in the already low numbers of reported faculty and PhD graduates disproportionately affect the academic *R*_0_. Finally, nonengineering PhD graduates getting professorships in engineering departments and vice versa could not be distinguished in this analysis.

## Supplementary Material

pgae169_Supplementary_Data

## Data Availability

The data underlying the results presented in the study are available on the ASEE website (https://ira.asee.org/by-the-numbers/).
