# Supplementary material for: Competition for engineering tenure-track faculty positions in the United States
Source: PNAS Nexus. 2024 May 7;3(5):pgae169. doi: 10.1093/pnasnexus/pgae169 (PMC11075531; doi:10.1093/pnasnexus/pgae169)
Supplement: pgae169_Supplementary_Data [file pgae169_supplementary_data.pdf]

**Supplementary Information**

# Competition for engineering tenure-track faculty positions in the United States

Siddhartha Roy*, Brenda Velasco and Marc A. Edwards

*Corresponding author email: siddhartha.roy@rutgers.edu

## This file includes:

Text S1

**Text S1.** Data discrepancy issue communicated to ASEE.

From: <sidroy@vt.edu>
Date: Sat, Jul 8, 2023 at 6:51 AM
Subject: ASEE annual reports -- possible data issue
To: XXX@asee.org
Cc: edwardsm@vt.edu <edwardsm@vt.edu>, Brenda Velasco <brendavelasco@vt.edu>

Dear XXXXXX –

I have been reviewing ASEE's annual reports for data on total TT/tenured faculty and came across a possible data issue. The total tenured/TT faculty count for 2019 is much higher than that for 2020 and 2021. I reviewed reports from pre-2019 years and it seems like there was a jump in 2019, which appears to be a data entry or analysis issue for certain engineering disciplines. Can you please review the below and give us correct 2019 data? We are trying to use these data for a peer-reviewed article.

Table 79: Number of Tenured/Tenure-Track Faculty by Engineering Discipline 50,586

Year 2019

| Discipline Name | Number of Faculty |
|---|---|
| Mechanical | 8,492 |
| Electrical/Computer | 8,097 |
| Comp Sci. (inside Eng.) | 5,322 |
| Chemical | 3,992 |
| Civil/Environmental | 3,472 |
| Biomedical | 3,123 |
| Civil | 3,009 |
| Other | 2,733 |
| Industrial/Manufacturing/Systems | 2,294 |
| Computer Sci. (outside Engr.) | 2,168 |
| Electrical | 2,117 |
| Metallurgical and Matrls. | 1,734 |
| Aerospace | 1,341 |
| Engineering (General) | 1,286 |
| Biological and Agricultural | 952 |
| Computer | 566 |
| Engr. Science and Engr. Physics | 516 |
| Nuclear | 362 |
| Petroleum | 309 |
| Envr. Eng | 295 |
| Engr. Management | 232 |
| Mining | 206 |
| Architectural | 136 |

* Total does not include computer science (outside

https://ira.asee.org/wp-content/uploads/2021/06/Engineering-by-the-Numbers-2019-JUNE-2021.pdf Pg 55

Table 79: Number of Tenured/Tenure-Track Faculty by Engineering Discipline 29,003

Year 2020

| Discipline Name | Number of Faculty |
|---|---|
| Mechanical | 4,946 |
| Electrical/Computer | 4,349 |
| Comp Sci. (inside Eng.) | 3,182 |
| Chemical | 2,178 |
| Civil | 1,906 |
| Biomedical | 1,729 |
| Civil/Environmental | 1,673 |
| Other | 1,597 |
| Computer Sci. (outside Engr.) | 1,355 |
| Electrical | 1,298 |
| Industrial/Manufacturing/Systems | 1,218 |
| Metallurgical and Matrls. | 1,116 |
| Engineering (General) | 858 |
| Aerospace | 804 |
| Biological and Agricultural | 560 |
| Computer | 373 |
| Engr. Science and Engr. Physics | 258 |
| Envr. Eng | 238 |
| Nuclear | 231 |
| Petroleum | 171 |
| Engr. Management | 136 |
| Architectural | 99 |
| Mining | 83 |

* Total does not include computer science (outside engineering).

https://ira.asee.org/wp-content/uploads/2021/11/Total-by-the-Number-2020.pdf Pg 52

Table 79: Number of Tenured/Tenure-Track Faculty by Engineering Discipline 28,593

Year 2021

| Discipline Name | Number of Faculty |
|---|---|
| Mechanical | 5,023 |
| Electrical/Computer | 4,270 |
| Comp Sci. (inside Eng.) | 3,116 |
| Chemical | 2,110 |
| Other | 1,946 |
| Civil/Environmental | 1,784 |
| Civil | 1,720 |
| Biomedical | 1,702 |
| Computer Sci. (outside Engr.) | 1,293 |
| Electrical | 1,219 |
| Industrial/Manufacturing/Systems | 1,195 |
| Metallurgical and Matrls. | 961 |
| Engineering (General) | 818 |
| Aerospace | 686 |
| Biological and Agricultural | 497 |
| Computer | 340 |
| Engr. Science and Engr. Physics | 282 |
| Nuclear | 247 |
| Envr. Eng | 236 |
| Petroleum | 174 |
| Engr. Management | 109 |
| Architectural | 93 |
| Mining | 65 |

* Total does not include computer science (outside engineering).

https://ira.asee.org/wp-content/uploads/2022/11/Engineering-and-Engineering-Technology-by-the-Numbers-2021.pdf Pg 52

Many thanks,

Sid
